# Effects of Ultrasonic Scaling and Teeth Brushing on Surface Properties of PEEK Prosthetic Restorations

**DOI:** 10.3390/dj14050303

**Published:** 2026-05-15

**Authors:** Aleksandra D. Čairović, Mirjana M. Perić, Nevena Čairović, Luka Župac, Vesna M. Maksimović, Sanja S. Stevanović, Aleksandra B. Špadijer-Gostović, Dragan M. Stanimirović

**Affiliations:** 1 Department of Prosthodontics, School of Dental Medicine, University of Belgrade, Rankeova 4, 11000 Belgrade, Serbia; aleksandra.cairovic@stomf.bg.ac.rs (A.D.Č.); mirjana.peric@stomf.bg.ac.rs (M.M.P.); cairovicnevena2030@gmail.com (N.Č.); sanja.dent@gmail.com (A.B.Š.-G.); 2 Vinča Institute of Nuclear Sciences, National Institute of the Republic of Serbia, University of Belgrade, Mike Petrovića Alasa 12-14, 11000 Belgrade, Serbia; vesnam@vinca.rs; 3 Institute of Chemistry, Technology and Metallurgy, University of Belgrade, Njegoševa 12, 11000 Belgrade, Serbia; sanjas@ihtm.bg.ac.rs; 4 Department of Periodontology and Oral Medicine, School of Dental Medicine, University of Belgrade, Rankeova 4, 11000 Belgrade, Serbia; dc_dragan@yahoo.com

**Keywords:** BioHPP, surface roughness, AFM, XRD, wetting, ultrasonic scaling, teeth brushing

## Abstract

**Purpose:** This study provides a comparative evaluation of surface changes in BioHPP materials under routine professional hygiene procedures, which is recommended by dentists, twice a year. BioHPP is a polyetheretherketone polymer used in prosthetic dentistry as a frame material. The aim was to investigate whether routine dental cleaning procedures such as ultrasonic scaling and brushing affect the surface proprieties of prosthetic BioHPP restorations. This study was conducted to evaluate the surface properties of different restorations based on BioHPP (veneered with composite resin and polished) after brushing and ultrasonic scaling exposure. **Materials and Methods:** The BioHPP specimens were divided into three groups. The first group (marked BioHPP) served as a baseline reference for assessing the effect of different surface processing approaches, and no further treatment was applied. The specimens in the second group (BioHPP-P) were polished, while the specimens in the third group (BioHPP-C) were veneered with composite resin. Group BioHPP-P and BioHPP-C of samples was divided into three subgroups: 0—no treatment, 1—exposed to tooth brushing, 2—exposed to ultrasonic scaling. Untreated samples (subgroup 0) served as controls for evaluating treatment-related changes within groups 2 and 3. The surface morphology was investigated by atomic force microscopy (AFM). The structure of samples was analyzed using the XRD technique, and the surface wettability was evaluated. **Results:** The surface roughness of the samples was evaluated via root mean square (RMS) parameter. Baseline BioHPP specimens exhibited higher roughness values compared to the other analyzed groups. The roughness of the non-treated specimens (0) decreased in the line 59.18→28.84→14.51 nm. Treatment of the samples by brushing and ultrasonic scaling was associated with an increase in surface roughness. Variations in water contact angle values were observed. However, no consistent treatment-related trend could be established. **Conclusions:** Composite veneered BioHPP showed a tendency toward higher surface resistance to brushing and ultrasonic scaling. These findings should be interpreted within the limitations of an in vitro descriptive study.

## 1. Introduction

This study aims to provide clinical guidance regarding appropriate hygiene maintenance protocols for BioHPP based restorations.

Polyetheretherketone (PEEK) is a synthetic, linear, thermoplastic, polymetric material and has been used as a biomaterial in medicine for many years, especially in orthopedic surgery [1,2].

The monomeric unit of PEEK is ethereterketone and the reaction polymerizes dialkylation of bis-phenolate to form polyetheretherketone.

Compared to other materials used as restorative prosthetics, PEEK has a low modulus of elasticity (4 GPa), similar to that of bone. Therefore, this material better withstands functional stresses, reducing the occlusal forces that are transmitted to the permanent teeth [3].

Since the PEEK material has a grayish-white opaque color, there was a need for veneering with composite materials in order to achieve satisfactory esthetic criteria [4,5]. Prosthetic restorations should last a long time in the patient’s mouth, and in order to obtain a chemically inert surface, BioHPP is veneered or glazed [6,7].

PEEK materials are used as frame materials in dental prosthetics, where fillers (ceramic particles of size 0.3–0.5 µm) are added due to better mechanical resistance. It is a partially crystalline, synthetic material, thermoplastic, stable and insoluble. Unlike metal alloys, it does not release ions and does not corrode, so it is recommended for patients with metal allergies. This material also has one quality from a technical point of view, which is that it is easily polished to a high gloss, which makes it plaque non-adherent and resistant to pigments. From the physical-mechanical aspect, PEEK materials have a modulus of elasticity like bone and are subject to torsion like bone which makes them suitable for restorations of a larger range. Its high pressure resistance (3.6 GPa), fracture resistance (1200 N) and low specific weight (1.31 g/m^3^) are qualities that make BioHPP a better choice, compared to dental alloys and Zirconia.

BioHPP can be used in prosthetic dentistry for all kinds of prosthetic restorations and can be applied as pure, not polished (the inside surface of an internal telescopic crown), polished (removable partial dentures) or veneered with composite (external telescopic crowns), skeletal prosthesis hook, all esthetic kind of dental prosthetic restorations, implant supported fixed restorations, All on four complete dentures [8,9,10]. In recent years, PEEK materials have been improved by adding different fillers, depending on the purpose. In implantology, PEEK can be used as an implant body, implant abutment or as a superstructure. As a material in implantology, Peek has weaker osteoconductive properties than titanium, but this can be improved by the addition of various bioactive materials. In implantology, it appears that PEEK’s time is yet to come [11].

All of these dental restorations need to be cleaned by the patient, but also at the dentist. This paper should give us advice on what cleaning method to apply in order to cause no damage to the investigated PEEK material.

In order for this material to be used in dentistry, it had to meet certain biological and mechanical criteria. This was achieved by enhancing pure PEEK with ceramic particles (less than 0.5 µm in diameter) [12]. Modified PEEK polymer, reinforced with ceramic particles (BioHPP, Bredent, GmbH, Senden, Germany) has excellent mechanical properties, good wear resistance, chemical inertness and good biocompatibility [13]. Thanks to that, BioHPP^®^ is the only material that achieves an ideal balance between elasticity and stiffness [14]. The surface of this material can easily be technically processed to a high gloss surface, polishing down to <0.02 µm, resulting in less plaque adhesion [15]. This material also has one property that is important for clinical diagnostics: BioHPP is radiolucent and does not create imaging artifacts.

From an esthetic point of view, compared to dental alloys, PEEK materials have the advantage of being white in color. Also, they can be veneered with composites in order to achieve high esthetics. Dental composites are metal-free, multiphase materials consisting of an organic matrix, an inorganic filler and a binder. Inorganic fillers are dispersed inorganic particles.

Thanks to a color that is more similar to the color of the teeth than the color of metal alloys, its metal-free composition and its modulus of elasticity—which is similar to the elasticity of tooth dentin—PEEK has found wide application in dental prosthetics in fixed and removable dental prostheses, dental implants, implant-supported fixed prostheses, implant-retained overdentures and hybrid dentures [16].

The complete dental hygiene of the oral cavity involves the care of all natural teeth, soft tissues, and dental prosthetic restorations and any supporting implants. Patients with prostheses are more prone to tooth decay and periodontal infections due to the accumulation of biofilm present at the margins of the restorations and under the pontic elements [17].

For the longevity of prosthetic restorations, remaining teeth, and soft tissues, the most important thing is good and proper oral hygiene. Also, it is very important to know the type and material properties that the prosthetic restorations are made from.

Based on different kinds of prosthetic restorations, some of them can be removed from the oral cavity before the hygiene session, but some have to be instrumented within the oral cavity. In order to provide hygiene we use manual and mechanical instruments, such as curettes and ultrasound.

Ultrasonic scaling and brushing are a form of professional oral hygiene maintenance procedure that is carried out every 2–3 times a year. This measure prevents the formation of dental plaque and the growth of calculus.

There are many different procedures by which the dentist maintains the hygiene of teeth and prosthetic restorations, the most common of which are the use of a brush and professional abrasive paste and ultrasonic scaling. This paper aims to show whether these recommended dental procedures are harmless or can lead to damage to the surfaces of prosthetic restorations. More precisely, from this paper we should conclude and give advice on how careful and attentive the dentist should be in daily practice when performing the procedures of tooth cleaning and prosthetic restorations. These procedures were selected as they represent the most commonly applied professional hygiene methods in routine dental practice.

Surface characteristics of dental restorations, such as surface roughness and hydrophobicity, are associated with the adhesion of microorganisms [18,19]. It has been reported that rough restoration surfaces increase the accumulation of dental plaque and calculus, as well as microbial adhesion [20], which are directly related to the development of periodontal disease. There are numerous studies that prove the connection between surface roughness and biofilm adhesion [21]. In all these studies, the conclusion can be drawn that a rougher surface equals the accumulation and retention of more plaque. However, in most studies, materials that have been in use for many years were studied, unlike BioHPP, which is a newer-generation material and there is not much data on this topic in the literature. In particular, comparative data on the effects of routine professional hygiene procedures on differently processed BioHPP surfaces remain limited.

Consequently, the aim of this study is to determine whether routine brushing and ultrasonic scaling procedures affect the surface properties of polished and composite-glazed BioHPP prosthetic restorations.

Given the exploratory nature of this in vitro investigation and the high-resolution characterization techniques employed, the study was designed to identify comparative morphological and wettability trends rather than to perform statistical hypothesis testing.

The following surface characteristics of prosthetic restorations made of BioHPP material will be analyzed:

(i) surface roughness (SR),
(ii) wetting contact angle (WCA),
(iii) X-ray diffraction (XRD) analysis, before and after surface treatment by brushing and ultrasonic scaling.

The null hypothesis of this study was that different methods of professional oral hygiene (brushing and ultrasonic scaling) would not affect either SR, WCA or XRD values on the tested materials. Given the exploratory and descriptive nature of this study, the hypothesis was evaluated in terms of observed trends rather than statistical testing.

## 2. Materials and Methods

In this study, a PEEK material called BioHPP (Bredent Group (Senden, Germany)) was investigated. The name is derived from the abbreviation for Bio—biocompatible and HPP—high performance polymer. It is a thermoplastic material, which is used both in fixed and mobile prosthetics, for the production of combined restorations and restorations on implants.

Sample collection protocol of BioHPP samples:

A total of 66 specimens were prepared and divided into three material groups (*n* = 22 per group). In each group, one specimen had dimensions of 10 × 30 × 2 mm and was used for XRD analysis, while the remaining 21 specimens per group (10 × 10 × 2 mm) were used for surface characterization and wettability measurements. No formal sample size calculation was performed due to the exploratory design of the study.

Samples were manufactured in three ways [22]:

**1.** The first group of 22 samples (one sample with dimensions 10 × 30 × 2 mm + 21 samples with dimensions 10 × 10 × 2 mm) was obtained by pressing in the For2press apparatus(Bredent Group, Senden, Germany); in the following text, samples of this group will be designated as BioHPP.

The samples were first cut out of the wax block, in the specified dimensions.

The wax tiles thus obtained were placed in a cuvette and melted in a wax annealing furnace at 930 °C. After cooling the cuvette to 400 °C, granulate material BioHPP^®^ Bredent was introduced.

The material was melted for 10 min and then pressed in a For2press^®^ vacuum press (Bredent Group, Senden, Germany). The first group of samples was not further processed (no additional polishing).

**2.** The second group of 22 samples (BioHPP-P) was made by cutting in a CAD/CAM machine in the specified dimensions. The machine is VHF/K5^®^, five-axis simultaneous cutting (Ammerbuch, Germany).

All samples were processed with milling cutters for processing high-performance polymers (“Generation M”) and prepared for polishing. Tungsten carbide cutters were used, coated with a layer of diatite (a protective layer that reduces heating and vibrations).

The second group of samples was polished according to the manufacturer’s protocol, to a high gloss. The first step was treatment with an abrasive diamond brush (Abraso Fix, Avenue Miami, FL, USA). The second step is polishing with a horsehair brush (Rodeo) in combination with Acrypol paste. The third step is polishing with a goat hair brush in combination with Abraso-Star Glanz paste. The last step is polishing with a woolen brush to achieve a high gloss.

**3.** The third group of 22 samples (BioHPP-C) was veneered with composite. The samples were sandblasted with 110 µ aluminum oxide, at 2.5 Bar. Then they were cleaned and conditioned with Visio.link^®^ primer. The primer was applied and polymerized in a polymerization lamp, Bre.Lux^®^ power Unit for 90 s, on each sample. The nano-hybrid composite Crea.lign^®^ was applied to all samples from the third group.

The composite was polished according to a standard protocol to a high gloss [23,24].

All processing and polishing materials are Bredent^®^ products, as well as all used devices. The sample polishing procedure was carried out according to the protocol recommended by the manufacturer.

For clarity, BioHPP-P refers to polished samples, while BioHPP-C refers to composite veneered samples.

The second (BioHPP-P) and third (BioHPP-C) group of samples were divided into three subgroups:

1. No treatment (control samples).
2. Exposed to brushing (with a professional dental cleaning brush and abrasive paste, Super Polish, Kerr).
3. Exposed to ultrasonic scaling (with an ultrasonic scaler incorporated in a dental unit).

**Figure 1:**
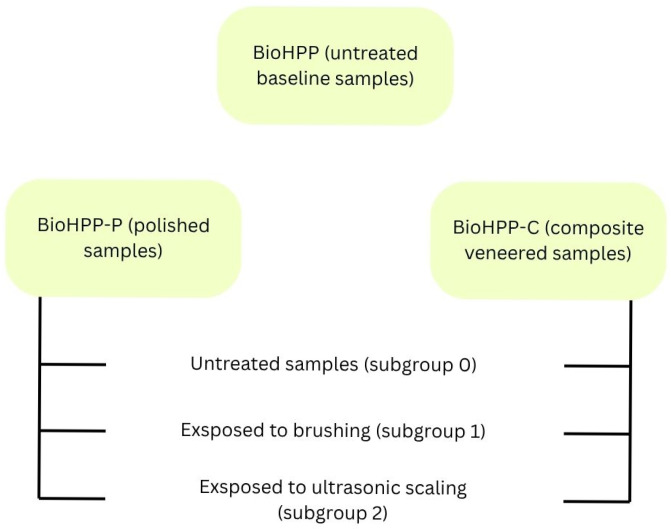
Schematic representation of the experimental protocol.

Untreated samples (subgroup 0) served as controls for evaluating treatment-related changes within groups 2 and 3 (Figure 1).

Application of brushing or ultrasound on each of the samples from the second or third subgroup of samples was for 1 min [25]. The exposure time was selected to simulate a standardized clinical intervention and to ensure comparability between treatment conditions. All procedures were performed under standardized conditions to minimize operator-dependent variability.

### Materials Characterization

The surface morphology was investigated by atomic force microscopy (AFM, HS, Amsterdam, The Netherlands) using a NanoScope 3D (Veeco, Plainview, NY, USA) microscope, operated in contact mode under ambient conditions. Silicon Nitride probes with spring constant of 20–60 N/m were used. Image analysis was done by NanoScope image processing software (V6.13). Image data was expressed in height mode. This approach allows the comparative assessment of morphological trends between the subgroups without relying on statistical inference.

The image roughness (RMS) is calculated as the root mean square average of height deviations taken from the mean data plane, where Z_i_ is the maximum vertical distance between the highest and lowest data points in the image:

$$\sqrt{\frac{\sum Z_{i}^{2}}{n}}=R_{q}$$

Considering the high-resolution and time-intensive nature of AFM and XRD analyses, one representative specimen per subgroup was selected for detailed surface topography analysis, which represents a limitation of the study. For each specimen, two scan areas (10 × 10 μm and 20 × 20 μm) were recorded to verify topographical consistency. Therefore, the obtained RMS values are presented descriptively, focusing on comparative morphological trends rather than statistical inference.

Phase composition of the representative samples (BioHPP, BioHPP-P 0,1,2 and BioHPP-C 0,1,2) was analyzed by XRD RIGAKU Ultima IV diffractometer, with Ni-filtered Cu Kα radiation (λ = 1.54178 Å). The X-ray diffraction data were collected over the 2θ range from 5° up to 80° with the step of 0.02° and scanning rate of 5°/min. All XRD analyses were performed on representative specimens from each subgroup to ensure consistent comparison of phase composition.

The wettability measurements were performed on a homemade device equipped with a CCD detector. During the measurements, a blue LED lamp (Kingbright Electronic Co., Ltd., New Taipei City, Taiwan) was used to illuminate the samples. Three samples from each group were randomly selected for the wettability measurements using a sessile drop method. All selected samples were measured in the same conditions: room temperature 22 ± 2 °C and relative humidity (not controlled). Each sample was evaluated by five drops of polar liquid-deionized water. After carefully dispersing a drop (2 μL) of water from a micropipette onto the surface at a 90° angle and from 5 mm distance, the photos were obtained 2 s after the drop touched the surface. The angle of the tangent to the surface of a water drop was measured using Drop Analysis software (V.3.11.3.1973). The obtained contact angles were analyzed descriptively to identify trends in surface wettability across the sample groups.

Accordingly, the overall study design was intentionally descriptive, aiming to provide high-resolution surface characterization and to compare treatment-related trends without statistical generalization. The results were analyzed descriptively to identify comparative trends.

## 3. Results

**Figure 2:**
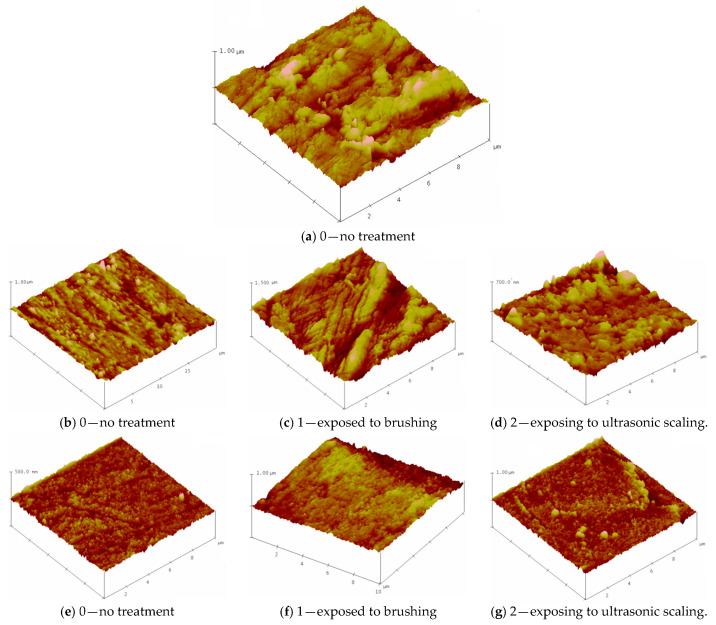
Atomic force microscope images of (**a**) untreated BioHPP, (**b**–**d**) BioHPP-P; (**e**–**g**) BioHPP-C.

Figure 2: depicts the three-dimensional morphology of the representative samples using AFM. Surface plot image displays the selected image with color-coded height information in a three-dimensional perspective.

The surfaces of the (a) BioHPP (b) BioHPP-P and (e) BioHPP-C specimens in the condition marked with 0 (no treatment), exhibited different morphologies. The three tested groups of samples were treated in different ways: the first group of samples was not polished after pouring, so the tested surface showed roughness as expected, in contrast to the second and third groups of samples, where the surface of the second group was coated with a composite and processed according to the protocol, until high gloss, and in the third group polished to a high gloss, also according to the manufacturer’s protocol.

The surface roughness of the samples was evaluated via root mean square (RMS) parameters, as shown in Table 1. The roughness of the non-treated specimens (0) showed a decreasing trend in the sequence 59.18→28.84→14.51 nm for the 10 μm × 10 μm scan area. The same trend occurs in the case of results for the surface plot image 20 μm x 20 μm (see Table 1). Treatment by brushing and ultrasonic scaling was associated with an observable increase in surface roughness values.

Relatively higher RMS values were observed after brush treatment compared to ultrasonic scaling in the analyzed specimens (samples exposed to brushing—BioHPP-P, 1 (101.82 nm), BioHPP-C, 1 (41.57 nm) and exposed to ultrasonic scaling BioHPP-P, 2 (36.9 nm), BioHPP-C, 2 (31.92 nm)).

**Figure 3:**
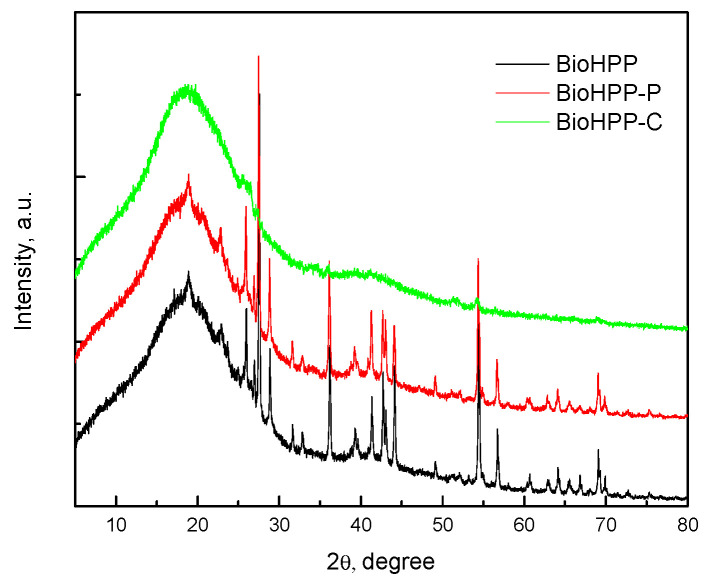
XRD patterns the representative samples; BioHPP (black); BioHPP-P (red); BioHPP-C (green).

Figure 3 shows the XRD spectrum of the BioHPP as well as BioHPP-P, 0 and BioHPP-C, 0. The broad diffraction peak, at approximately 2θ ≈ 18.5° corresponded to the characteristic peak of PEEK [26,27,28] and the peaks at approximately 2θ 25.8°, 27.5°, 28.8°, 36.3°, 54.5° and the others were the peaks of ceramic particles. The XRD spectrums of samples BioHPP-P, 1,2 and BioHPP-C, 1,2 are identical to XRD spectrums BioHPP-P, 0 and BioHPP-C, 0.

In order to assess the relative hydrophilic/hydrophobic properties of the BioHPP the water contact angle of the surface of the representative samples was determined. Table 2 represents the variation in the contact angle of various prepared samples.

Wetting angles with water were determined from the images given below, using Drop Analysis software.

**Figure 4:**
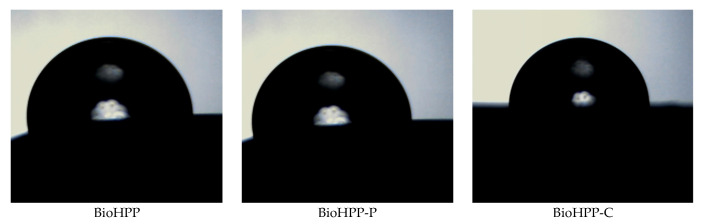
BioHPP, BioHPP-P and BioHPP-C samples wetted by water.

Different surface treatment influences surface morphology (Figure 4) and therefore wettabality. The water contact angle of the samples BioHPP-P, 0, 1, 2 and BioHPP-C, 0, 2 was >90° indicating a hydrophobic nature. On the contrary, the lowest contact angles and most hydrophilic surfaces were those on the samples BioHPP, 0 and BioHPP-C, 1, where the water contact angle was <90° [29].

These observations indicate that the wetting behavior is not only governed by surface morphology but probably also by the physical characteristics of the materials.

The wettability characterization is sensitive to detect very small variation in treatment, but no one can expect wettability tests to be used as a substitute for topographical analysis. In some cases, comparative tests may be envisaged [30].

The presented findings reflect descriptive comparative trends observed in representative specimens and should be interpreted within the exploratory framework of this investigation. No formal correlation analysis between surface roughness and wettability was performed due to the descriptive nature of the study.

## 4. Discussion

In everyday dental practice, professional teeth cleaning, as a routine procedure, is recommended every six months. Brushes and abrasive professional pastes combined with ultrasonic scaling are the most often used procedures for these purposes. This paper should give us an answer to the question of whether these recommended professional cleaning methods affect the quality of the surface of dental restorations made of BioHPP. Therefore, the following surface properties were analyzed: surface roughness, wetting angle and XRD analysis.

The results showed that there are differences in values of surface properties between the tested groups, as well as between subgroups, whose surface was treated differently. Within the limitations of this descriptive in vitro study, the observed trends appear not to support the initial null hypothesis.

It should be emphasized that these observations are based on descriptive high-resolution analyses of representative specimens and therefore reflect comparative trends rather than statistically validated differences.

An important factor in the prevention of alteration of the surface properties of prosthetic restorations is the polishing and final finishing process [31]; this represents one of the key observations of this study and from the literature data, as well.

Brushing with abrasive paste and ultrasonic scaling were associated with increased surface roughness values in all analyzed BioHPP material samples. There is evidence that brushing and ultrasonic scaling increase the surface roughness of many other restorative prosthetic materials such as ceramics, alloys, resins [32,33]. These findings are generally in line with previously reported trends for PEEK-based materials, although direct comparisons are limited due to differences in study design and methodology. Recent studies have demonstrated that similar surface modifications including mechanical treatments influence surface roughness and surface characteristics [34,35,36]. For example, various surface treatments such as sandblasting, polishing, and coating have been shown to alter surface properties.

The differences in the surface roughness values between the different BioHPP samples can be explained by the fact that the BioHPP samples veneered with composite make the surface mechanically more resistant and the composite covering the BioHPP surface may act as a protective layer. However, other factors such as material heterogeneity and variability in polishing procedures may also have contributed to the observed differences.

Polished forms of BioHPP (BioHPP-P-0,1,2) samples showed relatively higher surface roughness values and also deeper surface scratches after brushing with abrasive paste and ultrasonic scaling [37].

A rougher surface forms a greater adhesion surface for microorganisms and therefore may contribute to the formation of microbial biofilm [38]. The surface roughness of biomaterials highly affects bacterial responses, including their adhesion and spread. Generally speaking, smoother surfaces on a material better prevent bacterial adhesion and growth over its surface [39]. The results of the studies showed that the threshold value above the threshold of 0.2 µm of surface roughness affected the accumulation of bacteria or the formation of biofilm [40]. However, it should be noted that the RMS values obtained in this study are in the nanometer range, which limits direct comparison with the commonly cited micrometer-scale threshold for bacterial adhesion. Although increased roughness values were observed after brushing and ultrasonic scaling, all measured RMS values remained below the widely cited 0.2 µm (200 nm) threshold associated with enhanced bacterial retention. However, they were within the tongue detection threshold of 0.25–0.5 μm, except for BioHPP-P samples after ultrasonic scaling [41].

Studies show that the hydrophobicity of the material, as well as the high surface energy of the material, can affect microbial adhesion [42,43]. BiohHPP-C samples had larger contact angles and were considered to be more hydrophobic than BioHPP-P. Also, the samples after ultrasonic scaling had the highest wetting contact angles. The relationship between surface roughness, wettability, and biological response is complex and not always linear, as reported in previous studies [44,45]. Recent narrative reviews on PEEK-based materials, including BioHPP, have further emphasized that surface characteristics such as roughness, wettability, and surface energy play a key role in determining biological behavior and clinical performance [46]. Variations in surface chemistry, as well as micro and nanostructural characteristics, may contribute to inconsistent wettability behavior, which may explain the absence of a clearly defined trend in the present studies on dental biomaterials [47]. The underlying mechanisms governing these changes were not specifically investigated in this study.

XRD analysis provides recording of the diffraction or reflection of rays on solid (crystalline) surfaces. In this case, those solid particles represent particles of ceramic fillers that are added to BioHpp to improve the physical and mechanical properties of this material. The composite layer that covers one group of the tested samples represents a barrier to XRD rays and that is why ceramic particles present in deeper layers, under the composite, could not be detected.

Plaque adhesion on PEEK differs from that of other dental restorative materials such as ceramics and metals [48,49].

It has been confirmed that materials with a more hydrophobic surface facilitate the growth of hydrophobic bacteria. Candida albicans, which is generally considered to be the main cause of denture stomatitis, is considered to be hydrophobic, and this is an important factor for the initial adhesion of Candida albicans [50]. These interpretations should be considered speculative, as no direct microbiological analysis was performed.

The rough surface favors not only adhesion of dental plaque, but also easier discolouration. BioHPP coated with composite appears to have greater resistance to damaging caused by brushing and ultrasonic scaling, and therefore to the retention of microorganisms and discoloration. Bearing in mind that BioHPP represents a newer material, but also a material from which much is expected in the future, we are witnessing that there are more and more attempts to apply this material to implantology.

In daily practice, apart from brushing and ultrasound, other manual and mechanical instruments are used, such as, for example, steel, graphite and titanium curettes. Unlike manual instruments, the use of mechanical instruments is less dependent on the dexterity and skills of the dentist.

In this regard, it is of great importance to choose an adequate hygiene maintenance technique in order to maintain the optimal properties of the material. Polishing of the surface has a great influence on the durability of the color. Any mechanical damage also affects the increased accumulation of plaque and pigments from food and drinks, as well as smoking [51]. Aging, as a universal process, affects the color stability of the restorative materials in prosthetics, considering the fact that permanent restorations stays in the patient’s mouth for a long time. The color stability depends on the free surface energy and the roughness of the surface of the material, which is influenced by the surface polishing treatment. Many studies have proven that there is a positive correlation between surface roughness and discoloration [52,53,54]. Also, a better polished surface reflects more light than a rough surface, and this is also one of the important optical factors.

Clinical extrapolation of these findings should be approached with caution due to the in vitro and descriptive nature of the study.

A major limitation of this study is its descriptive design, including the analysis of one representative specimen per subgroup for AFM and XRD characterization. In addition, contact angle measurements were interpreted descriptively without inferential statistical analysis. Due to the high-resolution and time-intensive nature of these techniques, the present work should be considered exploratory and hypothesis-generating rather than statistically generalizable. Future investigations with larger sample sizes and quantitative statistical approaches are necessary to validate and expand upon these preliminary findings.

## 5. Conclusions

Within the limitations of this descriptive in vitro investigation, both brushing and ultrasonic scaling appeared to increase the surface roughness of BioHPP-based materials, with brushing appearing to have a more pronounced effect in the analyzed samples. Composite-veneered BioHPP appeared to show a tendency toward comparatively greater surface resistance to these professional hygiene procedures. These findings may have potential clinical relevance for the maintenance of BioHPP-based prosthetic restorations subjected to routine hygiene procedures and should be interpreted within the limitations of a descriptive in vitro study design. Further studies with larger sample sizes and statistical validation are required to confirm these observations.

## Figures and Tables

**Table 1:** Surface roughness (RMS values) of representative (analyzed) samples.

Samples	(Surface Plot Image 10 × 10 μm) RMS (nm)	(Surface Plot Image 20 × 20 μm) RMS (nm)
BioHPP	59.18	55.01
BioHPP-P, 0	28.84	29.43
BioHPP-P, 1	101.82	102.23
BioHPP-P, 2	36.90	35.98
BioHPP-C, 0	14.51	13.83
BioHPP-C, 1	41.57	40.34
BioHPP-C, 2	31.91	31.86

**Table 2:** Contact angles with water.

Sample	Mean Value	Sd
BioHPP,	88.6	4.38
BioHPP-P, 0	93.3	5.23
BioHPP-P, 1	93.3	1.98
BioHPP-P, 2	92.4	5.37
BioHPP-C, 0	92.65	0.92
BioHPP-C, 1	87.85	2.33
BioHPP-C, 2	102.00	3.96

## Data Availability

The data presented in this study are available on request from the corresponding author.
